# Comparison of Physiological and Perceptual Responses Between Continuous and Intermittent Cycling

**DOI:** 10.2478/v10078-011-0060-7

**Published:** 2011-10-04

**Authors:** Roxana M. Brasil, Ana C. Barreto, Leandro Nogueira, Edil Santos, Jefferson S. Novaes, Victor M. Reis

**Affiliations:** 1 Castelo Branco University, Rio de Janeiro, Brazil; 2 Celso Lisboa University; 3 Federal University of Rio de Janeiro, Rio de Janeiro, Brazil; 4 Department of Sport Sciences, University of Trás-os-Montes and Alto Douro (UTAD), Vila Real, Portugal; 5 Research Centre for Sports Sciences, Health and Human Development (CIDESD), Vila Real, Portugal

**Keywords:** cycling, training method, hemodynamic responses, blood lactate, perceived exertion

## Abstract

The present study tested the hypothesis that the exercise protocol (continuous vs. intermittent) would affect the physiological response and the perception of effort during aquatic cycling. Each protocol was divided on four stages. Heart rate, arterial blood pressure, blood lactate concentration, central and peripheral rate of perceived exertion were collected in both protocols in aquatic cycling in 10 women (values are mean ± SD): age=32.8 ± 4.8 years; height=1.62 ± 0.05 cm; body mass=61.60 ± 5.19 kg; estimated body fat=27.13 ± 4.92%. Protocols were compared through two way ANOVA with Scheffé’s post-hoc test and the test of Mann- Whitney for rate of perceived exertion with α=0.05. No systematic and consistent differences in heart rate, arterial blood pressure, double product and blood lactate concentration were found between protocols. On the other hand, central rate of perceived exertion was significantly higher at stage four during continuous protocol compared with intermittent protocol (p=0.01), while the peripheral rate of perceived exertion presented higher values at stages three (p=0.02) and four (p=0.00) in the continuous protocol when compared to the results found in intermittent protocol. These findings suggest that although the aquatic cycling induces similar physiologic demands in both protocols, the rate of perceived exertion may vary according to the continuous vs. intermittent nature of the exercise.

## Introduction

Physical training is often based on continuous and/or intermittent methods with the purpose of improving cardio respiratory ability (Barbosa et al., 2009). Studies have compared the effect of those methods on heart rate (HR) (Arngrimsson et al., 2003; Roecker et al., 2002), on arterial blood pressure (Mourot et al., 2004; Crisafulli et al., 2004), on double product (Forjaz et al., 1998), on blood lactate concentration (Gharbi et al., 2008; Sabapathy et al., 2004) and on the rate of perceived exertion (Borg and Kaijser, 2006; Lagally et al., 2002). However, these studies have not addressed the aquatic cycling. Cycling is one of the most popular means of exercise for general and specific physical conditioning and industry experts have adapted cycling equipments to the aquatic environment. Due to the nature of the equipment and of the aquatic environment, this type of exercise stands out as one more option for cardio respiratory training, applicable to different age groups and fitness levels by adapting the postures and grips the indoor stationary cycling.

According to Barbosa et al. (2009), the use of aquatic cycling has been reported in literature for three decades, though its findings are still contradictory. Alberton et al. (2010) suggest that HR in the water could be similar or higher as compared with dry land measurements. Barbosa et al. (2010) analyzed the relationships between musical cadence and the physiological adaptations to basic head-out aquatic exercises. The study included an intermittent and progressive protocol and the main conclusion was that increasing musical cadence imposed an increase in the physiological response. In this context, several physiologic indicators have been used in order to quantify the intensity of exertion in those environments, such as: the HR (Sheldahl et al., 1984; Reilly et al., 2003); double product (Veloso et al., 2003), and blood lactate concentration (Di Masi et al., 2007).

In water, resting or exercising induces different physiological responses when compared with those achieved in dry-land conditions (Shono et al., 2000; Reilly et al., 2003) and are affected by a number of factors, such as buoyancy, thermal conductivity of the water (Choukroun and Varene, 2000), hydrostatic pressure (Goodall and Howatson, 2008), among others. Those responses depend also on the body positioning in the water (Millet et al., 2002; Egaña et al., 2006) and on the type of exercise (Barbosa et al., 2009).

Kang et al. (2005) compared the responses of HR between intermittent (130 ± 2 bpm) and continuous cycling (127 ± 2 bpm) on land and did not found significant differences between both methods. The lactate concentration was significantly higher at the end of the intermittent exercise with a mean value above 7 mmol in the final stage of the IP. Contrarily, Sabapathy et al. (2004), have examined the physiological responses in 10 subjects who performed a continuous and intermittent land cycling protocol and observed that the intermittent protocol was associated to significantly lower values of HR. Unfortunately, no previous study examined the type of physiological response induce by continue or intermittent exercise in water environment. Therefore, the present study tested the hypothesis that the type of exercise (continuous vs. intermittent) would affect the physiological response and the perception of effort during aquatic cycling.

## Methods

### Participants

Ten women (values are mean ± SD: age=32.8 ± 4.8 years; height=1.62 ± 0.05 cm; body mass=61.60 ± 5.19 kg; estimated body fat=27.13 ± 4.92%) of low risk, practicing regular classes of cycling in water for at least six months, participated in the study. All of them signed a written informed consent to participate in the study and in accordance with the norms for accomplishment of research with humans established in the Helsinki Declaration of 1975. The experimental procedures were approved by the Ethics Committee of the Institution.

### Procedures

All volunteers attended the test venue on three different days, with an interval of seven days. The volunteers were instructed not to workout exhaustively in the previous 24 hours; to remain well hydrated in the previous 24 hours and to avoid eating, smoking, drinking alcohol or caffeine three hours before the tests, as well as to sleep between 6 and 8 hours in the night before testing.

The volunteers were submitted to an anthropometrical evaluation, consisting of body mass and height measures (Filizola, PL150-Personal Line, Brazil). The same technician obtained all anthropometric measurements, on the right side of the subject’s body. Skinfold thickness was obtained with a Lange skinfold caliper. A 3-site skin fold equation for woman was used to estimate body density (Jackson and Pollock, 1978) and body fat was subsequently calculated using the Siri equation (Heyward and Stolarczyz, 2001).

To evaluate the cardio respiratory capacity, the individuals were submitted to the Balke protocol (1959) accomplished in a standard cycloergometer (Monark 868E, Monark-Crescent, Varberg, Sweden) in a laboratory setting. It was applied progressive loads of 25 W every two minutes, until reaching the maximum voluntary exhaustion (Balke, 1959). The volunteers were submitted randomly to two cycling sessions using the aquatic bicycle (Hydrorider, A1S1316, Italy). Both sessions had a total duration of 31 minutes with a seven days interval in between, and were always carried out at the same time of the day. The temperature of the pool water was between 30 and 31ºC and 50% of relative humidity. The level of the immersion in water on sitting position was at the xifoid process.

The exercise protocols had a total duration of 31 minutes and were divided in five stages. Tables 1 and 2 present respectively the characteristics of the Continuous Protocol (CP) and the Intermittent Protocol (IP). The pedaling cadence was controlled by a metronome (Yamaha, QT-1, USA). In position 1 the individuals remained seated with hands on the base of the bicycle handlebar; in position 2, standing up with hands on the base of the bicycle handlebar; and in position 3 and standing up with hands on the extremity of the bicycle handlebar. Cycling cadence was maintained throughout all testing between 80 and 100 revolutions per minute.

### Data Collection

Absolute heart rate (HR) was continuously measured with a cardio-frequency meter (POLAR®, A1, Finland) device and Rate of Perceived Exertion (RPE) was measured at the end of each minute of exercise (Borg Scale-CR10). Blood pressure (BP) and blood Lactate concentration (BLC) were also assessed in the last minute of each stage. For BP, was measured by auscultation technique, non-invasive, using a sphygmomanometer (Tycos®, CE0050, USA) and professional stethoscope (Marshall®, Omrow Health Care, USA). Capillary (finger) blood sample were collected for BLC with an YSI 1500 analyzer (Yellowsprings, OH, USA). Prior to each testing, the lactate analyzer was calibrated with standard lactate solutions of 2.5, 5.0, 10.0 and 15.0 mmol·L-1 (Yellowsprings, OH, USA).

### Statistical Analysis

After identification of data normality (Shapiro-Wilk test), a two-way analysis of variance (protocol type; stages) was applied to check the effect in the variables selected. A post-hoc Scheffé test was used to describe possible differences among the variables. The Mann-Whitney test was used to compare the rate of perceived exertion between the respective stages. The study admitted a significance level α= 0.05. The data were processed using Statistical software (Statsoft, version 6.0, USA). Data s presented as mean ± standard deviation (SD).

## Results

**Figure 1:**
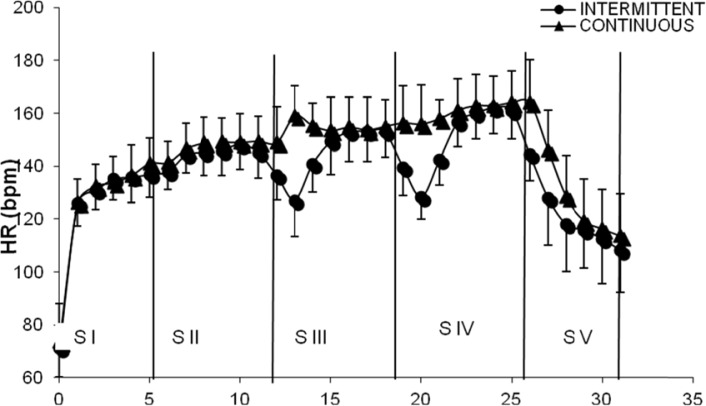
HR Time response in the intermittent (IP) vs. continuous (CP). Values are means ± SD. SI= Stage I; SII= Stage II; SIII= Stage III; SIV= Stage IV; SV= Stage V

The figure 1 shows the time on-response of HR response during both. Despite slightly higher values for continuous stages, most of the differences were not significant. In the second stage, when the individuals were pedaling standing up, the mean HR values during IP (149.00 ± 9.00 bpm) were significantly lower when compared with HR values during CP (158.00 ± 10.00 bpm).

**Figure 2:**
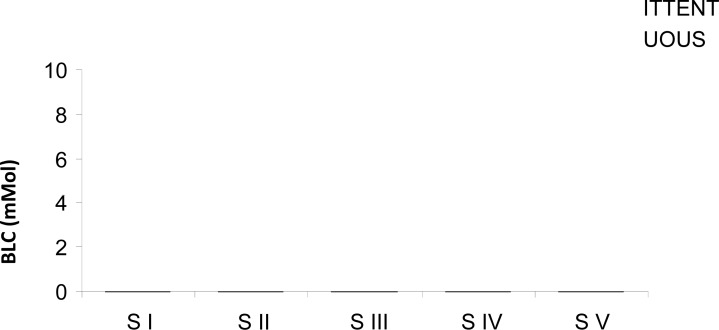
Blood Lactate (BLC) during each stage in the continuous (CP) and intermittent (IP) protocols. Values are means ± SD. * S I versus S IV in IP (P <0.05)

Significant differences were found in systolic blood pressure when comparing the mean values in Stage V of the IP (110.40 ± 13.78 mmHg) and of the CP (114.80 ± 13.70 mmHg) with the foregoing stages. The diastolic blood pressure, in the IP, Stage I (74.60 ± 6.11mmHg) presented a significant difference when compared with Stage II (66.40 ± 7.22 mmHg), with Stage IV (65.60 ± 8.04 mmHg) and with Stage V (64.60± 9.38 mmHg). Additionally, in the IP, Stage I (97.46± 7.54 mmHg) recorded mean values significantly higher than Stage V (79.86 ± 8.10 mmHg). Finally, in the CP, Stage I (94.13± 10.86 mmHg) showed a significant difference relatively to Stage V (82.93 ± 13.88 mmHg) (*P* < 0.05). The double product showed few significant differences between protocols. In the Intermittent Protocol significantly higher values were recorded in Stage I (19229.00 ± 3046.21 mmHg.bpm) when compared with Stage IV (22702.00 ± 232.05 mmHg.bpm). In the Continuous Protocol mean values observed in Stage I (18810.00 ± 2993.71 mmHg.bpm) were statistically different from those in Stage III (22408 ± 3687.04 mmHg.bpm) and in Stage IV (24345 ± 4641.42 mmHg.bpm). Figure 2 presents lactate concentration in the blood. The mean values observed at Stage I was significantly lower when compared with those at Stage IV in the continuous and intermittent protocols.

**Figure 3:**
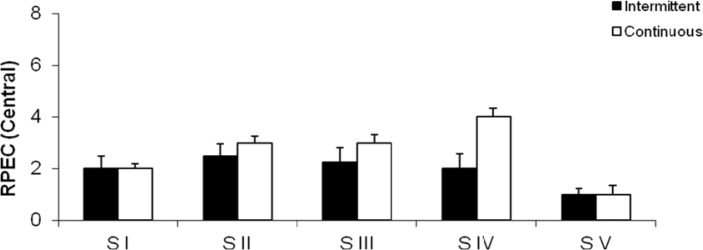
Central rate of perceived exertion (RPEC) at the end of each stage in the continuous (CP) and intermittent (IP) protocols.

**Figure 4:**
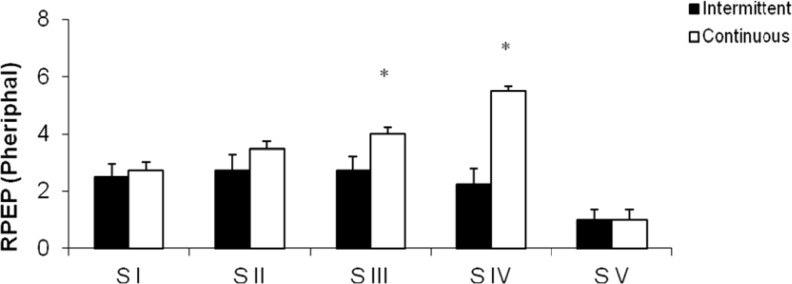
Peripheral rate of perceived exertion (PPEC) at the end of each stage in the continuous (CP) and intermittent (IP) protocols. *Significant difference between protocols (P <0.05).

Figure 3 and 4 show the mean rate of perceived exertion. In Figure 6 the differences for Central (RPEC) were not significant, in Stage I (CP:2.00± 0.47; IP:2.00± 0.96), Stage II(CP:3.00± 0.78; IP:2.50± 1.12), Stage III(CP:3.00± 1.05; IP:2.25±1.36) and Stage V (CP:1.00± 0.38; IP:1.00±0.23) except in Stage IV where the CP values (4.00±0.89) were higher than those found in the IP (2.00±1.18). In Figure 7 the results for Peripheral (RPEP) were significantly higher in Stage III (4.00± 0.89) and Stage IV (5.50± 0.95) in CP when compared to IP, respectively (3.00± 0.96 *vs.* 4.00± 1.33). However, the Stages I (CP:2.75 ± 0.77; IP:2.50± 1.01), Stage II (CP:3.50± 0.86; IP:2.75± 1.40) and Stage V (CP:1.00± 0.38; IP:1.00±0.38), all of them didn’t show significant values.

## Discussion

We have hypothesized that the short recovery periods in the IP combined with the aquatic environment would lower the physiological stress and perception of effort when compared with CP. The findings in this investigation demonstrated that heart rate (HR), double product (DP) and rate pf perceived exertion (RPE) were higher during the CP when compared with IP. However, blood pressure (both systolic and diastolic) and blood lactate presented higher mean values in IP when compared with CP. The RPE tended to be higher at the continuous protocol.

The HR response can be influenced by factors as: mechanical distension of the atrium, entailed by venous return, body temperature (Gastinger et al., 2010) blood acidosis (Knight-Maloney et al., 2002; Di Masi et al., 2007), environmental conditions (Arngrimsson et al., 2003) and the training method (Morris et al., 2003; Sabapathy et al., 2004). Kang et al. (2005) when comparing HR responses between intermittent (130 ± 2bpm) and continuous training methods (127± 2bpm) did not found significant differences between the two methods. The values by Kang et al (2005) were lower than those in the present study which can be explained by the lower elative exercise intensity (around 68% of HRmax). Since we have found differences in the HR between the two exercise protocols, we may suggest that these differences are more likely to occur the higher the exercise intensity involved. The findings from Kruel et al. (2009) seem to confirm this hypothesis. They analyzed the oxygen uptake, the heart rate and the energy expenditure of young active women in two routines of water exercises: continuous and interval. For all variables, significantly higher values were found in the interval routine.

In the present study the values of the BP, both systolic and diastolic, tended to be higher in IP, which could be justified by the fact that the individuals remained in the seated position during one and a half minute (corresponding to the recovery period), thereby probably increasing the hydrostatic pressure and stimulating the outlying baroreceptors, which could have facilitated the blood distribution and stimulated the venous return. Other than the position of the body, also buoyancy phenomena could have influenced our results (Sherman and Michaud, 1997). These findings confirm the study by Morris et al. (2003), who suggested that the intensity and the duration of the work series as well as the recovery period can influence the physiological responses and that these responses could be associated with the blood volume at the end of the diastole, which would induce an increase in the systolic volume through the Frank-Starling mechanism. In addition, as the BP and HR are directly influenced by the intensity of the exercise and also during the recovery period it is possible that the latter is responsible for the different effects observed in the DP in the present study. Ferreira et al. (2005), when comparing the DP between dry-land and aquatic cycling, found values between 17528.60± 1054.20 mmHg.bpm and 32697.00± 4136.26 mmHg.bpm, respectively in the two exercise conditions. Those values are higher than the ones in the present investigation. However, whereas we have studied adult women, the sample of the previous study comprised men. Therefore, we may suggest that factors as metabolic and hormonal levels, blood flow, and heart size (Carter et al., 2003) and body composition might have interfered in the different responses between studies.

Kang et al. (2005) reported a significant increase in blood lactate at the end of the IP when compared with CP, exceeding 7mmol/l at the end of the exercise protocol. We have failed to observe significant differences, though our mean values at the end of stage IV (the period of peak intensity) were also close to 7mmol/l in both protocols. Edwards et al. (1973) found values of 1.78± 0.55 and 5.77± 0.97mmol/l; 1.35± 0.37 and 4.07± 0.85 mmol/l, respectively during the IP and CP protocols at 25% and 50% of peak exertion in a dry-land cycle ergometer. Such values are lower than those observed in the present study. It can be inferred that different variations in the concentration of this metabolite may be related with the type of fiber, with the concentration of the protein transporters of the membrane, with the blood flow and its distribution and with the thermo conditions (Evertsen et al., 2001; Billat et al., 2003; Kang et al., 2005). Although not measured in our study, such factors could have also influenced our subjects and help to explain the differences between our study and that by Edwards et al. (1973).

The RPE was also addressed in the study by Kang et al. (2005), and it showed mean values of 8.90± 0.50 in the IP and of 9.70± 0.70 in CP. These values are higher than the findings of the present study, though they did not found significant differences between IP and CP. Contrarily we have found significant differences in RPE between the two protocols in stages III and IV. Several factors are believed to influence RPE, such as the exercise intensity (Alberton et al., 2010), the water depth (Di Masi et al., 2007), the water temperature (Fujishima and Shimizu, 2003) and the stimulation of peripheral receptors located on members and trunk (Robertson et al., 1995). The lower mean value for RPE in our study compared with those found in dry-land cycling. Kang et al. (2005) confirm that the aquatic environment may serve as a lowering factor in the perception of effort. Moreover, the fact that we found lower RPE mean values in IP when compared with CP suggests that the aquatic environment may enhance the effects of the short recovery periods involved in the intermittent exercise. It is important to consider on future studies could be performed with other population, other intensities, different aquatic environment such as temperature and depth.

The hypothesis that the intermittent protocol would offer a lower physiologic demand was only partially confirmed. HR, DP and RPE were higher during the CP. Plus, SBP and DBP and BLC presented higher mean values in the IP. The IP induced a more pronounced HR reduction and BLC during the recovery periods, suggesting a faster recovery in the intermittent protocol. More studies are recommended with other possible exercise intensity and volume combinations, as well as with different gender and conditioning level of the individuals.

## Figures and Tables

**Table 1:** Ccontinuous protocol (CP)

**Stages**	Time	intensity % of HR	position
Stage I	5	Between 75% and 80% of HRmax	Seated position 1
Stage II	7	Between 80% and 85% of HRmax	Seated Position 1
Stage III	7	85% of HRmax	Standing Position 2
Stage IV	7	Up to 92% of HRmax	Standing Position 3
Stage V	5	55% of HRmax	Seated

HRmax = maximal HR assessed in the Balke protocol; bpm = beats per minute

**Table 2:** Iintermittent protocol (IP)

**Stages**	Time	Intensity % of HR	Position
Stage I	5 minutes	Between 75% and 80% of HRmax	Seated Position 1
Stage II	5′ and 30″	Between 80% and 85% of HRmax	Seated Position 1
Recovery	1′ and 30″	Up to 75% of HRmax	Seated
Stage III	5′ and 30″	85% of HRmax	Standing Position 2
Recovery	1′ and 30″	Up to 75% of HRmax	Seated
Stage IV	5′ and 30″	92% of the HRmax	Standing Position 3
Recovery	1′ and 30″	Up to 75% of HRmax	Seated
Stage V	5 minutes	55% of HRmax	Seated

HRmax = maximal HR assessed in the Balke protocol; bpm = beats per minute
